# Perception of Remote Learning by Fixed Prosthodontic Students at a Romanian Faculty of Dentistry

**DOI:** 10.3390/ijerph20043622

**Published:** 2023-02-17

**Authors:** Oana Tanculescu, Alina-Mihaela Apostu, Adrian Doloca, Sorina Mihaela Solomon, Diana Diaconu-Popa, Carmen Iulia Ciongradi, Raluca-Maria Vieriu, Ovidiu Aungurencei, Ana-Maria Fatu, Nicoleta Ioanid, Mihaela Scurtu, Catalina Iulia Saveanu

**Affiliations:** 1 Discipline of Fixed Prosthodontics, Department of Odontology–Periodontology and Fixed Prosthodontics, Faculty of Dental Medicine, Grigore T. Popa University of Medicine and Pharmacy, 700115 Iasi, Romania; 2 Discipline of Medical Informatics and Biostatistics, Department of Preventive Medicine and Interdisciplinarity, Faculty of Dental Medicine, Grigore T. Popa University of Medicine and Pharmacy, 700115 Iasi, Romania; 3 Discipline of Periodontology, Department of Odontology–Periodontology and Fixed Prosthodontics, Faculty of Dental Medicine, Grigore T. Popa University of Medicine and Pharmacy, 700115 Iasi, Romania; 4 Discipline of Dental Technology, Department of Implantology, Removable Dentures, Dental Technology, Faculty of Dental Medicine, Grigore T. Popa University of Medicine and Pharmacy, 700115 Iasi, Romania; 5 Discipline of Pediatric Surgery and Orthopedics, 2nd Department of Surgery, Faculty of Medicine, Grigore T. Popa University of Medicine and Pharmacy, 700115 Iasi, Romania; 6 Discipline of Orthodontics and Dental-Facial Orthopedics, Department of Surgery, Faculty of Dental Medicine, Grigore T. Popa University of Medicine and Pharmacy, 700115 Iasi, Romania; 7 Discipline of Ergonomics, Department of Implantology, Removable Dentures, Dental Technology, Faculty of Dental Medicine, Grigore T. Popa University of Medicine and Pharmacy, 700115 Iasi, Romania; 8 Discipline of Preventive Dentistry, Department of Surgery, Faculty of Dental Medicine, Grigore T. Popa University of Medicine and Pharmacy, 700115 Iasi, Romania

**Keywords:** dental education, remote learning, online learning, COVID-19, practical skills

## Abstract

The COVID-19 pandemic has forced the transfer of traditional on-site educational activities to the online environment. This study aimed to evaluate the perception and acceptance of remote learning among fixed prosthodontic students attending the Faculty of Dental Medicine of “Grigore T. Popa” University of Medicine and Pharmacy in Iasi, Romania, and to analyze the feedback regarding their experience with the new online methods, the perceived quality thereof and suggestions for improvement. An observational cross-sectional, online study based on 22 questions was conducted with 259 students. The general opinion of online education was good or very good (40.15%); regarding its efficiency, 28.57% found it efficient while 34.36% found it inefficient or very inefficient; regarding the pleasure of learning online, 45.95% of students enjoyed online learning, while 36.64% did not enjoy it. The problem that was most cited by respondents was that of keeping all students motivated and involved (65.6%). Sixty-two percent of the respondents believe that online dental education should not exist, or just to a small extent, a result justified by the practical nature of the profession. The general opinion was that health risks should be managed and mitigated by using a hybrid system that would allow students to do on-site clinical training with direct contact with patients.

## 1. Introduction

WHO Director-General Tedros Adhanom Ghebreyesus declared, on 30 January 2020, the global outbreak of novel coronavirus to be a public health emergency of international concern (PHEIC) [1], the WHO’s highest level of alarm. On 11 March 2020 he declared that the rapidly spreading coronavirus should be considered a pandemic [2,3]. On 13 March 2020, Europe had already become the epicenter of the pandemic, with more reported cases and deaths than the rest of the world combined, apart from the People’s Republic of China [4]. Since then, due to the nature of the coronavirus, the entire world has experienced an unprecedented situation, with huge impacts on education and on health systems. The physical distancing and movement restriction of people has become the norm all around the world [5], consecutively impacting universities, regardless of the study programs [6,7,8]. Thus, the most common measure has been to transfer on-site classes to emergency remote teaching and, in some particular cases, online learning [9], keeping the students in protected environments until the pandemic conditions allow a safe return to face-to-face classes [10,11,12]. Though learning through online systems is not completely new, this sudden paradigm shift came with the need for a rapid and sustained adaptation for both students and faculty staff [12,13].

For dental faculties in particular, this change meant the transposition of practical work, performed on real patients, into the online environment. Dentistry requires close proximity of the doctor to the patient’s mouth, during therapeutic maneuvers, that generate aerosols which are incriminated in virus spreading [14,15,16,17,18,19]. Consequently, The US Centers for Disease Control and Prevention (CDC) declared dental care-related aerosols and droplets as high risk, considering the apparent resemblance between these aerosols and those specific to medical maneuvers, such as anesthesia or tracheal and nasopharyngeal procedures [15,20]. Therefore, the National Health Commission of China (NHCC), National Health Service of England and American Dental Association (ADA), followed by other dental health regulatory bodies, recommended dental care only in emergency situations during the COVID-19 outbreak period [21,22].

In this context, the dental educators scrambled to adjust an education of a practical and skill-based nature to the online environment. Though various platforms and methods are available for online teaching [23], many of these are of limited use or cannot be employed in the area of dental education [24,25].

For theoretical training, collaboration tools as Microsoft Teams^®^ (Washington, DC, USA, Microsoft Corporation), Zoom^®^ (San Jose, CA, USA, Zoom Video Communications, Inc.), Jitsi^®^ (Campbell, CA, USA, 8 × 8 Inc.), WebEx^®^ (San Jose, CA, USA, Cisco Systems, Inc.) and Moodle^®^ (Perth, Australia, Moodle HQ) were adopted by many universities, for conferences and lectures [23]. The major challenge was the practical preclinical and clinical dental training, for which some limited options are available including virtual reality-based technology, virtual patients, and dental training mannequins, all of which may be supported by lecture-based learning (LBL), problem-based learning (PBL), case-based learning (CBL), team-based learning (TBL), and research-based learning (RBL) [26,27,28]. Despite the general effort, there has been a high level of concern regarding the impact of these changes on student instruction [29].

In Romania, the bachelor’s degree of dentistry (B.D.S) program comprises six years of formal education and is divided into two parts i.e., a pre-clinical training stage (first and second year) and a clinical training stage (third to sixth year). Training programs in dental clinical skills are woven longitudinally into the preclinical curricula starting with the third year, when the students begin to work in clinical environments, on real patients, under the supervision of specialist doctors. Thus, the third and the fourth year are considered the beginning of clinical training and the fifth and the sixth year are the final years of study, consisting predominantly of clinical activities. Grigore T. Popa University of Medicine and Pharmacy of Iasi (UMPh Iasi) is a higher education institution, comprising four faculties: Medicine, Pharmacy, Dental Medicine and Biomedical Engineering. The faculties of Medicine and Dental Medicine have Romanian, English and French programs [30].

On 16 March 2020, the UMPh Iasi announced that all classes would be conducted completely online and remotely, with the cancellation of all on-campus learning, and hands-on and clinical training. The online content of courses and labs was available on the existing e-learning platform of the university. Microsoft Teams^®^ was implemented at the institution level for sending didactic material, conducting classroom conferences and lectures, posting videos, assigning tasks and assessments, and ensuring communication between students and professors. Customized online exams were used, with final hands-on assessment removed. 

**Figure 1:**
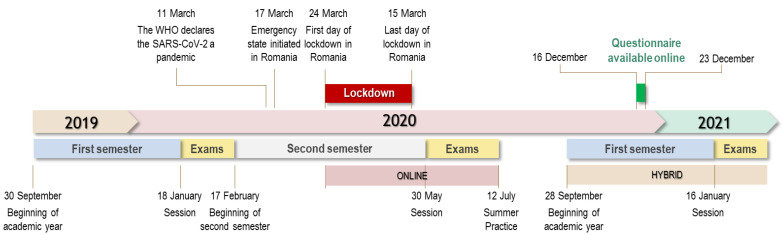
Timeline of academic activity due to the pandemic.

The 2020–2021 university year was hybrid—the courses being online and the labs on-site (Figure 1), keeping the clinical training on real patients at a minimum. This took into account the existing risk of infection through the various mutations of the virus, despite the high vaccination coverage among the students and teachers [31].

Fixed prosthodontics is one of the main fields of dentistry. At the UMPh Iasi, for the third, fourth and sixth-year students it is a leading compulsory subject of 7, 8 and 5 European Credit Transfer System (ECTS)points, respectively (344 teaching hours).

Modern dental education needs to keep up with the constantly growing knowledge in the biomedical sciences field and involves real-life situations, interpersonal interactions with patients, practice-based learning and the gaining of clinical experience. These are the main pillars of the curricular for dental clinical disciplines aiming to improve students’ psychomotor skills and knowledge in diagnosis and treatment option and planning.

In Romania, as in many other countries [23,28,32,33,34,35,36] during the pandemic period, concerns were raised regarding clinical internships at dental clinics [19,37,38]. Clinical training was deeply affected and each medical specialization, including fixed prosthodontics, tried to cope and to find solutions for knowledge transfer and for the compensation for the lack of clinical skills training.

The aim of this study was to evaluate the perception and acceptance of remote learning among fixed prosthodontic students attending our Faculty of Dental Medicine, and to analyze the feedback regarding their experience with the new online methods, the perceived quality thereof and suggestions for improvement.

## 2. Materials and Methods

### 2.1. New Teaching Methods

Microsoft Teams is a digital platform that centralizes content, assignments and communication tools in one place. MS Teams is well suited as a university-level virtual environment for education. It engages students with virtual face-to-face communication and activities, and, during the pandemic, it was the next best thing to classical onsite training.

In the first step, the university IT department created MS Teams accounts for all the students and the teaching staff. In this phase, several training sessions were organized so that everyone involved could quickly get familiar with the basic features of MS Teams and could start using the platform for daily educational activities (creating teams, assigning students, uploading content, creating meetings, creating assignments etc.). The selection of this collaboration platform was made based on the features that it offers, but also on the fact that the existing university IT infrastructure is also Microsoft-based and, as such, the integration was a natural process. The adoption of this new educational tool by the students was also a quick and easy process, as MS Teams has some resemblance to the already popular Skype. Members of the teaching staff were given the responsibility to create the necessary teams, mimicking the already existing student group structure and series. MS Teams is complementary to the existing university e-learning platform which provides not only learning content to the students but is, at the same time, an education management tool which registers attendance, student grades, announcements, notifications etc.

### 2.2. Sample and Questionnaire

An observational cross-sectional, questionnaire-based online survey regarding the remote learning system in use during the COVID-19 pandemic was conducted during 16 December and 23 December 2020. The study focused on students that were supposed to undergo clinical training at a fixed prosthodontics clinic, but the pandemic context drastically limited direct contact between them, the patients and all other persons involved in the educational process. These were students in the third, the fourth and the sixth year of study at the Romanian section of the Faculty of Dental Medicine at the Grigore T. Popa University of Medicine and Pharmacy Iasi. 

The questionnaire was created in Romanian and was setup using Google Forms (Mountain View, CA, USA, Alphabet). Misleading questions, multiple negations or unclear formulations were completely avoided. The questionnaire was reviewed for face validity by three experts in dental medical education in order to identify relevant key issues for dental medical students and to assess its relevance and accuracy. Additionally, the study was validated in a pilot study on 32 students. Their feedback and suggestions were used for improvement of the survey. None of these students participated in the final study.

The invitation to participate in the survey and the link to the Google Forms informed consent and questionnaire documents were posted online in Microsoft Teams, in all three teams corresponding to the involved clinical years, for all 488 Romanian students [39]. 

The representative sample size for the total number of third, fourth and sixth-year students (*n* = 488) was calculated for a confidence level of *p* = 95%, z = 1.96, and margin of error of 5%. The resulting calculated sample size was 216 [40]. Six hundred fifty students were enrolled in the clinical training years (third to sixth year). For the same confidence level (*p* = 95%) and margin of error (5%) the calculated sample size was 242. The questionnaire was answered by 259 students, representing 53% of the total number for the three targeted years. The sample was also representative for all clinical training years. 

Participant sampling was volunteer based, and no incentives were used for study participation. All respondents delivered answers to all questions in the questionnaire, making the acquired data valid and usable as provided. No data were eliminated. 

The questionnaire focused on students’ perceptions and feedback on didactic activities during pandemic period and was structured into three parts. The first part included single and multiple-choice general questions regarding remote learning and its impact (Q1–Q12). The second part also included multiple-choice questions related to the perceived quality of remote teaching, learning and assessment in the fixed prosthodontics disciplines for both theoretical knowledge and practical skills (Q13–Q20). Finally, the third part included two open questions, asking for suggestions to improve didactic activity for fixed prosthodontics disciplines and also for free comments on this subject. 

The first eight questions (Q1–Q8) had answers rated on a five-point Likert scale, representing ordinal variables, with different constructions of the answers, and each question was attached to the response scale with the corresponding coding. The response categories for these questions are presented in Table 1. The lower the score, the stronger is the negative perception of the student and the higher the score, the stronger is the positive students’ perceptions and acceptance of remote learning and its consequences.

The questions 9–20 (Q9–Q20) were purely nominal, with no ranking of the possible answers, while the remaining two were open-ended questions (Q21, Q22).

### 2.3. Statistical Study

In the first stage of the statistical analysis, the construction validity was tested using factors and reliability analysis, i.e., Cronbach’s alpha test. Descriptive statistical methods were used, determining frequencies for categorical responses and the distribution diagrams of these responses. 

The interrelation between some categorical variables was analyzed using contingency tables. To check if there is a statistically significant relationship between these variables, a chi-square test was performed. To assess the strength of the relationship, for ordinal variables, the Spearman’s rank correlation coefficient was determined. For nominal variables, the strength of the association was analyzed using Cramer’s V.

The answers to the open-ended questions were evaluated qualitatively and if there were more than two similar statements then they were placed into groups. This helped to understand the student needs and prioritize the measures needed to improve the quality of e-teaching and e-learning.

Data analysis was carried out using IBM SPSS Statistics, version 28 (IBM, Armonk, NY, USA). Statistical significance was set at *p* = 0.05.

## 3. Results

### 3.1. Student Perception and Feedback on Remote Learning and Its Impact

The compilation of the questions and their internal consistency (Q1–Q8) was tested and the reliability for each latent variable used in this study was confirmed by Cronbach’s alpha test (α = 0.829). Table 2 shows the parallel correlations between the variables, which are generally weak, with only six correlations above 0.500.

**Figure 2:**
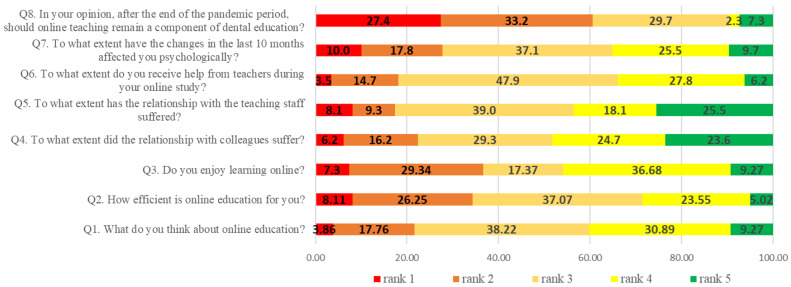
Distribution of relative frequencies of answers to questions one to three (Q1–Q8). The numbers in the bars indicate the percentages of the answers received (*n* = 259).

The first three questions are related to the students’ general opinion about online education (Q1), their perception about its efficiency (Q2) and the pleasure of learning online (Q3). One hundred four students (40.15%) have a good or a very good opinion about online education (Q1), while 99 (38.22%) are neutral and 56 (21.62%) have a bad or very bad opinion about online learning. Regarding the efficiency (Q2), only 74 (28.57%) found online learning to be efficient, while 89 (34.36%) found it efficient or very efficient. Of the students, 45.95% (119) enjoy online learning, while 36.64% (95) do not enjoy it (Q3) (Figure 2). Pearson’s chi-squared tests showed a strong correlation between the three items (Q1, Q2 and Q3) (*p* < 0.001) (Table 2).

Fifty-eight (22.4%) participants stated that their relationship with their colleagues suffered considerably, a percentage to which we add those who felt a moderate alteration of their relationship (29.3%) (Q4). It seems that not only the relations with colleagues have suffered but also those with the teaching staff (Q5). Fifty-six, 4% of the participants in the study claimed that this type of relationship suffered, from a moderate to an extreme intensity, especially in the conditions in which 17.4% of students perceived the support that they received from their teacher to be absent or very reduced (Q6). However, at the same time, the vast majority (81.9%) of students regard the help from teaching staff as moderate to extremely helpful. Of the students, 35.2% were not psychologically affected at all or only slightly affected, while 37.1% perceived moderate psychological changes (Q7). Having the experience of all types of learning—exclusively onsite, exclusively online and hybrid—62% of the respondents believe that online education should not exist, or just to a small extent, a result justified by the practical nature of the profession and by the university profile (Q8) (Figure 1).

**Figure 3:**
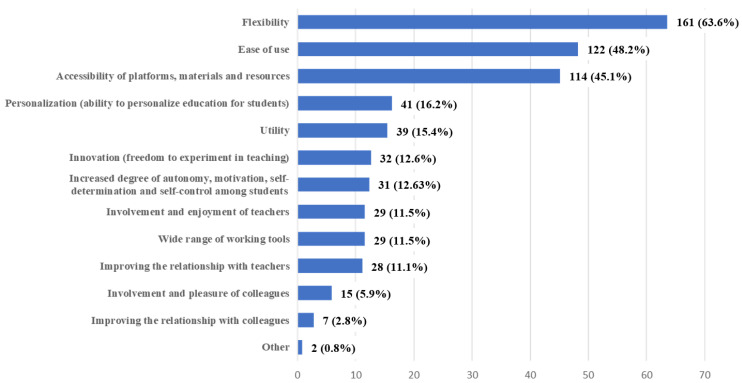
Frequency distribution of answers to question 9 (Q9): “What has pleasantly surprised you about online/remote learning?”. The numbers in the bars indicate the counts and the percentages of the answers (*n* = 259).

Q9 focused on the possible motivations for enjoying online/remote learning. The respondents were pleasantly surprised primarily by the high degree of flexibility offered by this type of education (63.6%), its ease of use (48.2%) and the accessibility of platforms, materials and resources (45.1%) (Figure 3).

**Figure 4:**
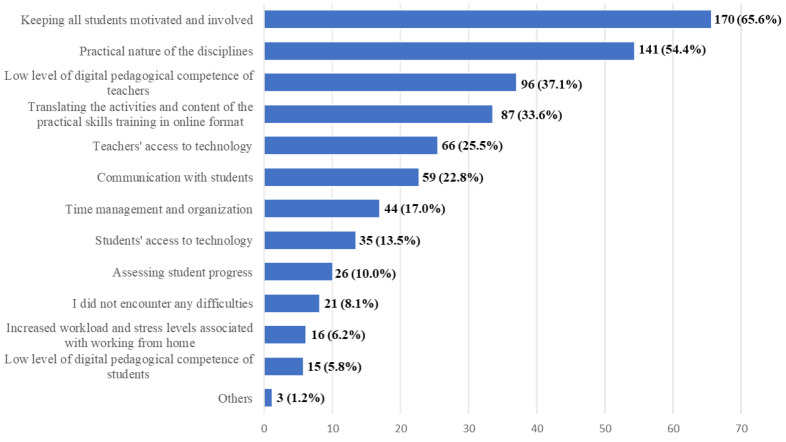
Frequency distribution of answers to question 10 (Q10): “In your opinion, what were the main obstacles to the transition to online/remote learning? Select up to five options”. The numbers indicate the counts and the percentages of the answers (*n* = 259).

The transition to online/remote learning (Q10), in addition to the need for immediate implementation, has encountered several other obstacles. According to the majority of dental students, the main challenges that were encountered were keeping all the students motivated and involved (65.6%) and the practical nature of the discipline (54.4%) (Figure 4). The low level of digital pedagogical competence of the teachers and the difficulty of translating the practical training to the online environment, were two other important obstacles claimed by the students.

**Figure 5:**
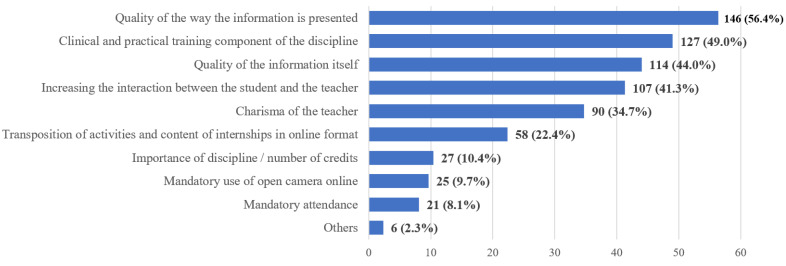
Frequency distribution of answers to Q11: “In your opinion what could increase your involvement during online activities?”. The numbers indicate the counts and the percentages of the answers (*n* = 259).

More than half of the students (56.4%) found the quality of the presentation to be the main instrument to increase student involvement during online/remote activities (Q11). The clinical and practical training component of the discipline is another factor which motivates the students to stay involved and focused (49%), followed by the quality of the information itself (44%) and an increase in the level of interaction between the students and the teacher (41.3%) (Figure 5).

**Figure 6:**
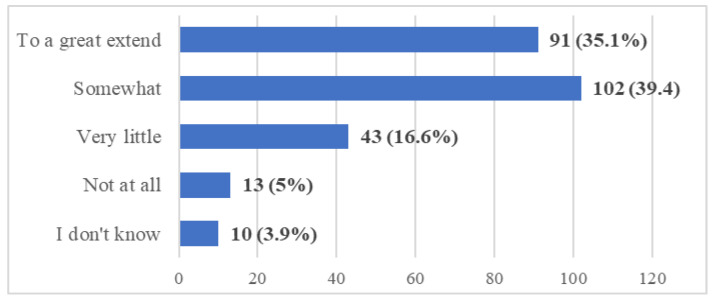
Frequency distribution of answers to Q12: “In your opinion, will the COVID-19 crisis change the future of dental education?”. The numbers indicate the counts and the percentages of the answers (*n* = 259).

Of the students, 74.5% are convinced that dental education will be changed due to the COVID-19 crisis (Q12) (Figure 6).

### 3.2. Student Perception and Feedback on Remote Teaching, Learning and Assessment at the Fixed Prosthodontics Disciplines for Both Theoretical Knowledge and Practical Skills

The major challenge related to online teaching has been the conveying of practical notions and clinical procedures to students, activities that traditionally require the presence of a patient. However, equally, we were interested in students’ opinions on the theoretical aspects of the courses, in terms of the manner of presentation—online, onsite or hybrid—and the personal interactions with the academic staff.

Regarding the preference for teaching theoretical notions—online, onsite or hybrid (Q13)—the respondents have equally divided opinions between hybrid and online. In the case of the online version, they prefer an increased share of activities carried out synchronously, with real-time interaction with the teaching staff. In their opinion, the teacher should personally present the course and thus reduce the need to watch recordings or video demonstrations with pre-recorded explanations (Q14) (60.23%) (Table 3).

**Figure 7:**
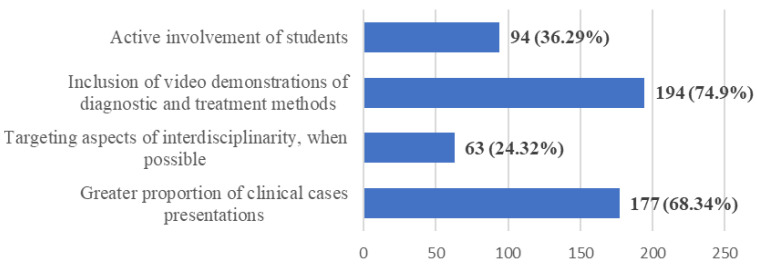
Frequency distribution of answers to Q15: “Do you consider that in teaching the theoretical notions the following should be considered:”. The numbers indicate the counts and the percentages of the answers (*n* = 259).

A very large proportion of students also desired (Q15) the inclusion, along with the pure theoretical notions, of a greater number of video demonstrations of diagnostic and treatment methods (74.9%), as well as an increased share of clinical cases (68.34%), with more active involvement of students during the course (36.29%) (Figure 7).

**Figure 8:**
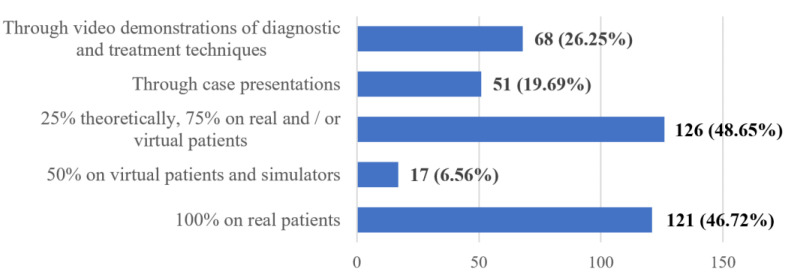
Frequency distribution of answers to Q16: “Do you consider that the teaching of practical knowledge should be carried out:”. The numbers indicate the counts and the percentages of the answers (*n* = 259).

Regarding the practical notions (Q16), there is an obvious preference for teaching using real patients, in a proportion of 100% (46.72%), or 25% theoretically and 75% on real and/or virtual patients (48.65%) (Figure 8). 

**Figure 9:**
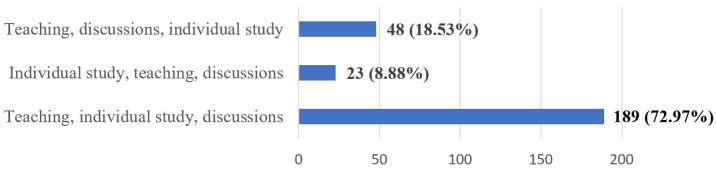
Frequency distribution of answers to Q17: “What do you think should be the sequence of steps?”. The numbers indicate the counts and the percentages of the answers (*n* = 259).

Most students (72.97%) prefer the following sequence of steps in the learning process (Q17): teaching, individual study and discussions (Figure 9).

**Figure 10:**
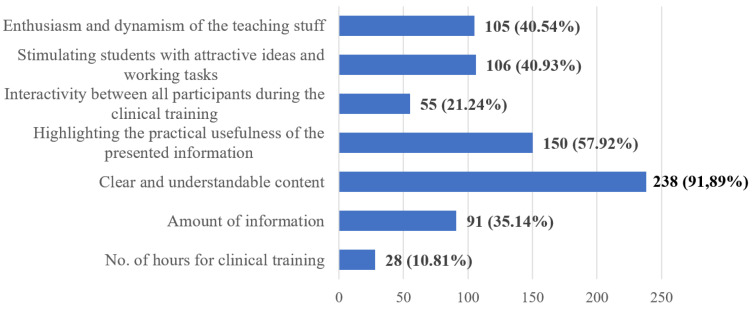
Frequency distribution of answers to Q18. “What are, in your opinion, the top three factors that influence the quality of clinical training?”. The numbers indicate the counts and the percentages of the answers (*n* = 259).

Q18 is related to the factors that could increase the quality of the clinical training considering the online conditions. The main factor is clear and understandable content (91.89%), along with a specifying of the practical usefulness and relevance of the received information (57.92%) (Figure 10).

**Figure 11:**
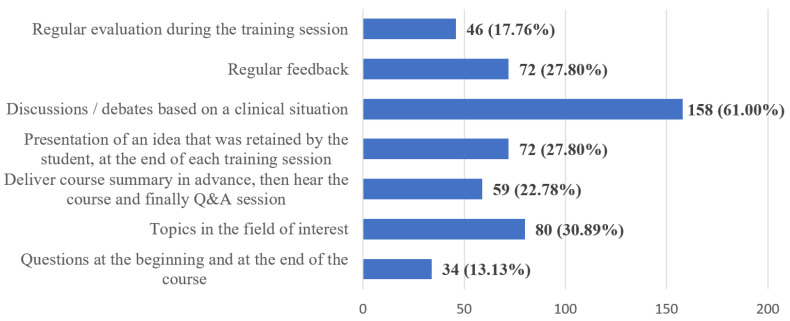
Frequency distribution of answers to Q19: “What are, in your opinion, the most appropriate methods for the optimal assimilation of the conveyed practical and theoretical notions?”. The numbers indicate the counts and the percentages of the answers (*n* = 259).

According to the students, the best way to facilitate the assimilation of the transmitted practical and theoretical notions (Q19) is through discussions and debates based on clinical situations (61.00%) (Figure 11).

**Figure 12:**
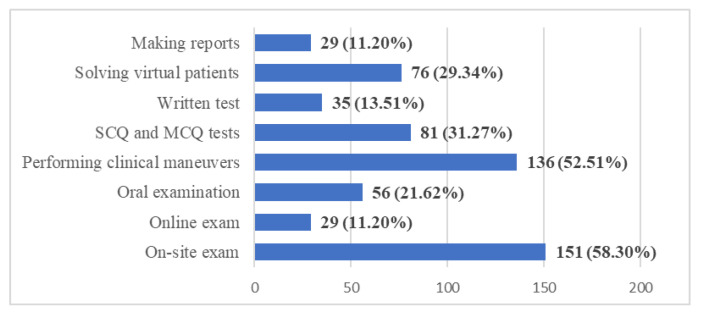
Frequency distribution of answers to Q20: “What are, in your opinion, the most appropriate evaluation methods, for the predominant practical/applied disciplines?”. The numbers indicate the counts and the percentages of the answers (*n* = 259).

Regarding the most popular method of examination/evaluation for disciplines with a practical component (Q20), students would prefer onsite examination (58.3%), with the performing of practical maneuvers (52.51%) and, in smaller proportion, the simple and multiple choices tests (31.27%), or use of virtual patients (29.34%) (Figure 12).

### 3.3. Open-Ended Questions–Students’ Suggestions and Opinions on Didactic Activity at the Fixed Prosthodontics Disciplines

The responses to open-ended questions (Q21: “What suggestions do you have for improving the teaching activity? Specify which of the disciplines you are making the recommendations for” and Q22: “Additional comments are welcome. Please note them down”) revealed the strengths and the limitations of online learning and some of the concerns and suggestions of our students, related to this new teaching approach (Table 4).

Some of the most frequent comments related to the strength points were the high attendance of online lectures, with the possibility of spending more time in a safe environment with family and with lower costs. The accessibility of digital content not otherwise available in the classrooms is perceived as an improvement to the teaching process. The most critical observation was that dental education is inherently an activity that must develop practical skills and cannot be performed only at a theoretical level or without any direct contact with the patient. Another frequent problem claimed by the students is the difficulty of keeping their focus and motivation to get involved during the online lectures. From these statements some concerns are derived, including insufficient abilities and training in patient treatment, and insufficient knowledge of the new online assessment method. The common suggestion for improving the educational process was to switch back to on-site teaching. Online teaching is accepted as a temporary solution given the pandemic situation, but one that is not suited for permanent adoption because of the many disadvantages. These ideas are summarized in one of the students’ answers: “I consider myself one of those focused and engaged students and I’ve still had classes to which I couldn’t pay attention at all. I don’t think the problem is the professor. They all worked very hard. The problem is that it is done online.”

## 4. Discussion

Due to its high level of transmissibility and casualties and to the imposed restrictions associated with it, the COVID-19 pandemic has disrupted almost every area of human activity. Education, which relies on people getting together to share knowledge, has been especially affected, making most of the usual activities impossible. Despite all these restrictions and challenges, dental schools around the world have quickly and creatively adapted to the new situation, to ensure continuity of the educational processes [11,12,13,19,38,41,42]. Grigore T. Popa University of Medicine and Pharmacy Iasi, similar to other universities, already had a digital platform that supported day –to-day academic activities: digital material sharing, management of absences and exam results, teaching staff evaluations, etc. However, the platform neither supported real-time communication between students and teaching staff, nor real-time management of the student periodic evaluations. This means that students were accustomed to the digital platform, mainly for administrative tasks, but the educational component was 100% a traditional one, with lectures given in amphitheaters and practical activities that took place in clinics. Therefore, the accelerated transition to a new, online way of teaching and evaluating was a huge challenge, both for teaching staff and for students [12,13].

Even if these days, the pandemic seems to be fading, we must recognize that the gains in the usage of new digital technologies and new teaching methods are expected to continue even after the pandemic is over [41,43,44]. Most of the dental schools have already made the transition back to face-to-face teaching, but the new methods used during the pandemic can and should complete and enhance the classical approach [45,46].

Our study is focused on the fixed prosthodontics disciplines and students, highlighting the needs and expectations for this domain.

Looking at the frequency distributions of answers provided to the Likert scale questions, we can extract a few interesting insights. Most of the answers are in the middle of the scale, avoiding very bad and very good statements. This is due to the realization that we are dealing with an exceptional situation, and we are trying to cope with it, doing our best. As an exception, answers to questions about the extent to which the relationships with the colleagues and with the teaching staff were affected, had a higher frequency towards the favorable end of the scale, stating that they were not at all affected. This is mainly due to the fact that, even prior to the pandemic, a lot of digital communication tools were available and which students used privately: Skype, WhatsApp, Messenger, etc. This ensured that they could stay in contact and not suffer from total isolation. These findings are also supported by a recent study made in different dental schools in the European region [47]. In addition to this, given the potential for psychological problems among students and academic staff induced by personal, social, emotional and academic situations [6,12,33,46,48,49], the Grigore T. Popa University of Medicine and Pharmacy Iasi offered guidance and emotional support on its online platform, via professional counseling sessions [33,50,51].

Another question with a high frequency of answers, this time towards the unfavorable end of the scale, is about the remaining online teaching methods after the end of the pandemic period. This clear negative reaction was caused not only by the difficulties posed by the new online teaching method and the new tools that have to be used, but also by the fact that without direct contact with patients and without hands-on practice, the quality of the educational process will significantly decrease [11,12,36,43,52,53]. In this regard, the biggest challenge for lecturers and professors has been to compensate for the lack of clinical training, and to re-invent and re-adapt the educational process in a very short period of time, without any previous preparation or planning, and while also constrained by distance, legal and ethical problems (such as the use of “show and share” patient clinical pictures in digital environment) [23,36].

When it comes to the positive sides of online teaching and learning, students have appreciated the flexibility, the ease of use and the potential for innovation. This shows that the introduction of new technologies and digital tools was not, by any means, a problem, but something that students embraced with lightness although it required some effort on their part as shown in our research and reported in different other studies [11,12,36,52,54,55].

If we were to pinpoint just one major issue linked to the transition to online/remote learning, this would be the lack of motivation and involvement in activities performed online. This aspect shows up numerous times through the questionnaire and seems to be the main hurdle affecting the new way of working during the pandemic [23,33,36]. Based on the experience of teaching staff with online lectures, an increased student attendance is not necessarily relevant, if their focus and engagement are low or, in some cases, even very low. Lack of visual contact with students, interference of home-related activities and interaction with family members, and difficulty in setting clear boundaries between the personal and professional space and schedules, usually account for disruptions of students’ concentration on the presented topics and for low engagement levels [48]. Our study tried to further investigate the ways in which this problem could be alleviated, and the possible solutions indicated by the students revolve around the quality of the presentations, the level of interaction between students and educators and around the possibility of more focus on the clinical and practical aspects of the presented information.

Given the pluses and minuses of the online/remote didactic activities, the majority (75%) of the participants in this study expressed their belief that the future of dental education will be impacted by the COVID-19 crisis. This is a general belief that has been revealed by many studies [9,10,12,23,29,36,42,44,45,46,48,50,53,54,56].

The type of teaching (hybrid/online/onsite) of the theoretical notions in relation to the content type was another evaluated issue. The outcome underlines the preference for online or hybrid teaching, with the distinction between the two being decided by the type of content—synchronous or asynchronous. The correlation between the two variables is a significant one. This is somehow expected because synchronous content requires real-time interaction with the teaching staff, while asynchronous content can be delivered fully online, without the instant supervision of an educator [36,57]. Overall, the students were more favorable to direct interaction with the professor, as it provides an opportunity to ask questions in real time and to get instant feedback [57,58]. In analyzing the online teaching of theoretical notions, the respondents identified two main possible ways of ensuring higher quality: using video demonstrations of diagnostic and treatment methods and including a higher proportion of clinical cases in the delivered presentations. These measures could, at least partly, compensate for the reduced level of interaction between the students and the educators and patients in the online environment.

Regarding the asynchronous teaching/learning, due to technological advancements and several undeniable benefits, dental podcasts have become popular among students and practitioners as tools for learning and for updating knowledge in general. Some studies [59] have shown that, in comparison with text book reading, watching video podcasts is a more efficient learning method, an efficiency that is reflected by higher scores in MCQ tests. Short-duration podcasts in particular [60] were perceived by students as useful supplementary learning tools that aided them for revision and in their preparation for assessments. An in-depth analysis of the importance of podcasts in learning in the medical field was undertaken by [61].

The value brought by this kind of teaching/learning practice is founded on several benefits that relate to the new modern way of living and working:

a. flexibility—being disconnected from the creation and transmission of the training material, the trainee can access it at any time, any place and using the preferred device. After downloading the material, some or all of the content can be played at the student’s discretion, so as to facilitate the learning process.
b. engagement—the content is more engaging than the mere reading of a textbook, further supporting the learning process.
c. wide accessibility—the content can be accessed anywhere in the world, thus reaching a much wider audience compared with on-site delivered lectures.

Regarding the teaching of practical skills, the general opinion is that this should be undertaken more on real patients and be less reliant on the use of video demonstrations and virtual patients. This is again linked to the fact that dentistry is regarded as a practical domain that should deliver education in a very practical way and in a setting that is as close as possible to a real clinical environment. Other alternatives are regarded as moving away from the normal path that education should progress on and could be accepted only because of the exceptional situation generated by the COVID-19 pandemic.

Though there is no replacement for hands-on clinical experience, versatile and immersive learning experiences can be obtained through haptic technologies and virtual (Moog Simodent) and augmented reality (DentSim, CDS-100, IRIS) [62]. These have the potential to deliver relevant, flexible, and immersive learning experiences if they are further enhanced according to the needs of dentistry. At the same time, they should be portable and affordable for large-scale usage by students [10,24,43,44,45,54]. Meanwhile, for medicine, some applications and software that are focused on complex, clinically based scenarios are available for use in virtual group discussions to improve students’ decision-making and diagnostic skills [26,63]. However, for dentistry in general, and prosthodontics, in particular, there are few options available for students [27].

Artificial intelligence (AI) is being increasingly adopted in the field of dentistry, including dental education. While other technologies, such as robotics, are expensive and require a certain environment in which to operate, AI can be delivered at much lower costs and to a wider range of students. AI can be used to provide virtual simulations of dental procedures, allowing students to practice and improve their skills in a safe and controlled environment. This can help mitigate the risk of errors or failures during real-life procedures.

Regarding the sequence of the learning activities, most of the respondents opted for teaching, individual study and discussions. The students considered it more opportune for the student–instructor and student–student interactions/discussions to be taken after the lecture series and individual study. This sets a high priority on individual study with the possibility to clear up some difficult subjects during discussions with members of the teaching staff [58].

Clinical training should be performed using clear and understandable content and focusing on the practical usage of the presented information, engagement of the students in different and attractive tasks, high interactivity between the participants to the study-group, and, not the least, by an enthusiastic and dynamic approach on the part of the professor. At the same time, the optimal assimilation of information should be supported through discussions/debates on relevant clinical situations, as pointed out by the students’ answers. Although these responses were not surprising, the implementation of these goals was difficult, the teaching staff were found to be unprepared for the translation of the clinical internships from the actual patient’s head to the online environment. It is worth noting that the literature highlights the general dissatisfaction of students regarding the organization of clinical internships, other online options being unable to compensate for the lack of actual practice [34,35,47,64].

The assessments are an important aspect of the educational process and were impacted as well by the pandemic. As educators, we tried to find out what the proper methods of assessment would be, given the challenges imposed by the fight against COVID-19. In our study, most of the respondents chose the on-site examination type, despite the risks related to their health. They opted for clinical maneuvers as a means to evaluate the assimilated knowledge, in the context of on-site evaluation. Simple choice questions (SCQ) and multiple-choice questions (MCQ) were also regarded as a possibility, being methods that are compatible with the available online tools. During the pandemic, universities have adopted several online assessment methods, including MCQ, oral examinations, video oral assessment, essays etc. While these are compatible with digital tools (MS Teams, Skype, Email), other problems arose: identity verification, authorship, and plagiarism, all of which might affect the validity and the relevance of the assessment [33]. Some of the universities or disciplines canceled or postponed the examinations [46], other universities adopted new procedures for the pandemic. Assessment of clinical competences was in some cases done using virtual patients, which could be a digital alternative to the face-to-face examination of clinical abilities. These specifically target assessment of clinical reasoning and of diagnostic skills.

Recent advances in AI have produced systems such as OpenAI’s ChatGPT and Google’s Bard, based on optimized language models and capable of interacting in a conversational way. This technology offers multiple opportunities for education, but it still must be seen how it can be best applied to specific needs and what adjustments are necessary so that it is used in a responsible way. The main goal is to improve and enhance the learning process while minimizing the risk of plagiarism and fraud related to assignments and exams.

Among the limitations of this research were the way in which the survey was conducted at only one dental institution, the study participants were self-selected and some of the questions focused on fixed prosthodontic disciplines, with clinical skills curricula. Another limitation of this study was the low response rate of the dental students (53%), which may contribute to non-response bias since targeted dental students were underrepresented. Based on the example of one department in the university and one single university in the country, the study cannot be positioned as a reference point for the actual situation. However, the authors consider that sharing experience is important and each university can consistently contribute to a new and improved teaching/learning paradigm. Student feedback is a valuable instrument in shaping curriculum and this is why their perspective and acceptance of the encountered transformations during the pandemic period have been previously reported in many studies [11,12,25,28,34,35,36,47,55]. Future research should be performed so as to also involve the teaching staff, to reconcile both views regarding the educational process.

At the same time, revolutionary technologies such as robotics and artificial intelligence (AI) could also contribute to the enhancement of the way dental education is delivered [65]. A study that looks at the acceptance and the impact of these novel methods among dental students would be an important tool that might help to integrate them along the traditional teaching practices. 

According to the results of this study, clinical training was the major challenge during pandemic crises. Despite the high acceptance of new digital tools, not all students embraced online learning, since there is a need for consistent enhancement of these tools to support clinical training. In the context of the limited availability of virtual or enhanced reality, proper digital tools (virtual reality-based technology, virtual patients, PBL, CBL etc.) can improve clinical reasoning and decision making. Still, for practical skills training, direct contact with the patient is mandatory. None of the other available methods were able to compensate for the lack of practical skills training. During the pandemic we put an accent on clinical reasoning and decision making which was an improvement compared with previous years. In our case, as a consequence of the feedback gained from this study, we had to adjust accordingly and implement, over the following years, the recovery of practical skills training in fixed prosthodontics. Constant feedback from students and flexible curricula structures might be powerful instruments in adapting the educational tools to student needs. 

On one hand, the curriculum must be flexible enough to accommodate a wider range of learning tools that include digital applications which can be used, if needed, without a physical presence. On the other hand, it is necessary to expand these tools to stimulate clinical reasoning and decision making remotely, without physical contact. Additionally, for the improvement of dental clinical skills and beyond the traditional simulators, enhanced and virtual reality systems with haptic feedback are required, though these entail high costs and require special equipment. 

## 5. Conclusions

COVID-19 was a global force majeure event that imposed drastic measures on all levels of human existence: healthcare, education, economy, and social life. For dental education in particular, the disruption imposed by this pandemic revealed once more the importance of direct contact of students with teaching staff and with patients. Regardless of the progress in computer technology used for online teaching, there is a unanimous opinion that dentistry is not a domain that can be predominantly taught online. However, online teaching can be a substantial addition to the traditional on-site method. This is due to several advantages that online teaching offers, including: high availability, usage of an enhanced variety of digital materials, independence of location and time, reduced costs for both students and universities. If we are to admit any positive sides of this tragic event, it would be a better understanding of the value of human interrelations and the accelerated progress of medical and computer technology, especially of the online communication platforms, telemedicine, and simulations. These technologies should be integrated into the educational process as an instrument to boost the trainer’s ability to engage students and improve their practical skills.

## Figures and Tables

**Table 1:** The response categories for questions one to eight.

Questions	Answers Rank				
	1	2	3	4	5
Q1. What do you think about online education?	very bad	bad	neutral	good	very good
Q2. How efficient is online education for you?	not at all	a little bit	medium	a lot	very much
Q3. Do you enjoy learning online?	not at all	not really	neutral	yes, but with some changes	yes, definitely
Q4. To what extent did the relationship with colleagues suffer?	very much	a lot	moderate	a little bit	not at all
Q5. To what extent has the relationship with the teaching staff suffered?	very much	a lot	moderate	a little bit	not at all
Q6. To what extent do you receive help from teachers during your online study?	very much	a lot	moderate	a little bit	not at all
Q7. To what extent have the changes in the last 10 months affected you psychologically?	very much	a lot	moderate	a little bit	not at all
Q8. In your opinion, after the end of the pandemic period, should online teaching remain a component of dental education?	no	to littleextent	moderate extent	to a largeextent	yes

**Table 2:** Spearman’s rank correlation coefficient for Q1–Q8 (N of valid cases = 259).

		Q1	Q2	Q3	Q4	Q5	Q6	Q7	Q8
Q1	*χ^2^*	-							
	*ρ*	1							
	*1-β*	-							
Q2	*χ^2^*	285.979	-						
	*ρ*	0.786 **	1						
	*1-β*	1	-						
Q3	*χ^2^*	230.655	210.226	-					
	*ρ*	0.635 **	0.708 **	1					
	*1-β*	1	1	-					
Q4	*χ^2^*	44.846	40.859	33.992	-				
	*ρ*	0.234 **	0.306 **	0.229 **	1				
	*1-β*	0.883	0.991	0.866	-				
Q5	*χ^2^*	69.652	118.120	69.142	192.041	-			
	*ρ*	0.418 **	0.490 **	0.359 **	0.586 **	1			
	*1-β*	1	1	0.999	1	-			
Q6	*χ^2^*	53.662	63.246	33.946	29.172	39.950	-		
	*ρ*	0.386 **	0.375 **	0.236 **	0.189 **	0.288 **	1		
	*1-β*	1	1	0.889	0.677	0.981	-		
Q7	*χ^2^*	51.571	72.787	50.956	57.135	57.083	48.482	-	
	*ρ*	0.334 **	0.421 **	0.330 **	0.332 **	0.358 **	0.318 **	1	
	*1-β*	0.998	1	0.997	0.997	0.999	0.995	-	
Q8	*χ^2^*	99.090	97.099	113.490	18.962	40.878	18.202	38.243	-
	*ρ*	0.493 **	0.536 **	0.590 **	0.168 **	0.282 **	0.063	0.230 **	1
	*1-β*	1	1	1	0.547	0.976	0.059	0.869	-

χ2 = Pearson’s chi-squared statistic for df = 16; ρ = Spearman’s rank correlation coefficient; 1-β = statistical power of the test. ** Correlation is significant at the 0.01 level (two-tailed).

**Table 3:** Spearman’s rank correlation coefficient for Q1–Q8 (N of valid cases = 259).

			Q13-Do You Consider That the Teaching of Theoretical Notions Should Be Done:								
	Answer Options		Hybrid	Online	Onsite	*χ^2^*	*df*	*ϕ_c_*	*p*	Total	
			***N***	***N***	***N***					***N***	***%***
Q14	Increasing the amount of asynchronous content		34	50	19	7, 873	2	0.17	<0.020	103	39.77
	Increasing the amount of synchronous content		65	49	42					156	60.23
	Total	N	99	99	61					259	
		%	38.22	38.22	23.55						100.00
Q14 = Do you think that the following should be considered when teaching theoretical notions online											

*N* = count; χ2 = Pearson’s chi-squared statistic; ϕ_c_ = Cramer’s V coefficient; *p* = *p*-value.

**Table 4:** Some of the most relevant student opinions.

Strengths	Limitations	Concerns	Suggestions
Unlike previous years, we managed to attend all the courses. More time to spend in a familiar, safe environment. Reduced costs for transportation and accommodation for students from outside the university center. Increased usage of digital teaching content during the classes. Availability of online teaching materials to be watched at any time.	Lack of practical skills. Dentistry is not a theoretical domain; hands-on practical training is mandatory. We were struggling to keep our concentration and motivation at a high level. Lack of interactivity with the peers and teaching staff. Not all the disciplines allow the courses to be recorded and do not offer online materials. Lack of separation between the work environment and home environment. Distraction by family-related issues in the home environment. Internet connectivity issues.	Because of the lack of practical activity, the information will be superficially assimilated, without a deep understanding. Lack of experience of patient interaction and treatment, with concerns about the future profession. Lack of social interaction and the alteration of the student–professor relationship. Stress and uncertainty related to the new online examination method, irrelevant and altered quality of online examination. Losing the privacy of one’s own home because of the need to turn on the camera and bringing the stress related to the faculty activities to the private environment at home.	In person attendance at the clinical training. The level of interactivity during the online classes should be increased. On-site examination. At least those who had COVID should attend on-site classes.

## Data Availability

The data that support the findings of this study are available on request from the corresponding author.
